# Using 2D qNMR analysis to distinguish between frozen and frozen/thawed chicken meat and evaluate freshness

**DOI:** 10.1038/s41538-022-00159-x

**Published:** 2022-09-22

**Authors:** Hyun Cheol Kim, Ki Ho Baek, Yee Eun Lee, Taemin Kang, Hyun Jun Kim, Dongheon Lee, Cheorun Jo

**Affiliations:** 1 Department of Agricultural Biotechnology, Center for Food and Bioconvergence, and Research Institute of Agriculture and Life Science, Seoul National University, Seoul, Republic of Korea; 2 Division of Chemical and Biological Metrology, Korea Research Institute of Standards and Science, Daejeon, Republic of Korea; 3 Surface Technology Division, Korea Institute of Material Sciences, Changwon, Republic of Korea; 4 Institute of Green Bio Science and Technology, Seoul National University, Pyeongchang, Republic of Korea

**Keywords:** Metabolomics, Metabolomics

## Abstract

We identified key metabolites reflecting microbial spoilage and differentiated unfrozen meat from frozen/thawed (FT) using 2D qNMR analysis. Unfrozen and FT chicken breasts were prepared, individually aerobically packaged, and stored for 16 days at 2 °C. Only volatile basic nitrogen (VBN) was significantly changed after 6 log CFU/g of total aerobic bacteria (*p* < 0.05). Extended storage resulted in an increase in organic acids, free amino acids, biogenic amines, and hypoxanthine and a decrease in N,N-dimethylglycine, inosine 5′-monophosphate, and proline. Acetic acid demonstrated the highest correlation with VBN (*r* = 0.97). Unfrozen and FT breast meat can be differentiated by uniform concentration of carnosine, β-alanine, and histidine levels, consistent changes in nucleotides by storage time, and changes in microbial metabolism patterns that are reflected by some free amino acids. Thus, NMR-based metabolomics can be used to evaluate chicken breast meat freshness and distinguish between unfrozen and FT meat.

## Introduction

With increasing incomes, meat and meat-based product consumption has been increasing worldwide because of its superior taste and nutritional value^1^. Chicken is a popular source of meat among health-conscious consumers due to the perception that it contains low fat and cholesterol content^2^. Chicken meat has a short shelf life because it is easily spoiled by microorganisms, which result in the deterioration of meat quality through the production of odorous end products such as volatile basic nitrogen (VBN) and lipid peroxides, which deteriorate the sensory quality of meat^3–5^. Because of its short shelf life, chicken meat is often stored frozen. However, frozen/thawed (FT) chicken has diminished quality, resulting in a lower price than unfrozen chicken^6^. Sometimes, FT chicken meat can be illegally mixed with unfrozen chicken and distributed when the product is in high demand^6,7^. FT meat is more susceptible to microbial spoilage owing to leakage of fluid that contains nutrients for microorganisms^8^ .Microbial contamination level is recognized as an important criterion for evaluating meat freshness and safety^9^. When microbial contamination occurs, metabolites are consumed differently, depending on the characteristic metabolisms of the microorganisms, resulting in different metabolomes that can be distinguished^10–12^.

Compared with other chemometric analyses, nuclear magnetic resonance (NMR)-based analysis has the advantages of short run times, simple preparation requirements, and good reproducibility^13,14^. NMR-based analysis is one of the leading types of analysis, along with mass spectroscopy. However, NMR analysis is inherently limited because it involves the measurement of a mixture without an analyte separation process, thus, the technique has a relatively lower resolution compared with that of mass spectrometry^15^. To overcome this limitation, high-magnetic-field NMR or two-dimensional quantitative NMR (2D qNMR) analysis can be used^16^. 2D qNMR-based metabolomics is an advanced method to elucidate the metabolic differences by comparing 2D qNMR results with those of one-dimensional proton NMR (1D ^1^H NMR) via expansion dimension, which allows the tracing of higher metabolites simultaneously without erroneous qualification that could be occurred in 1D ^1^H NMR analysis^17^. Furthermore, abundant metabolic information can be used to accurately identify key metabolites and differentiate between samples using discriminant analysis^18^.

The objective of this study was to identify key metabolites that represent microbial spoilage during storage and to differentiate unfrozen meat from FT chicken breast meat simultaneously based on metabolic information using 2D qNMR analysis.

## Results and discussion

### Total aerobic bacteria (TAB) and physicochemical properties

Microbial and physicochemical properties were measured to evaluate the freshness of chicken breast meat (Table 1). The FT breast meat had significantly higher microbial contamination than that in unfrozen meat (*p* < 0.05). The high microbial contamination levels of the FT breast meat seemed to result from the leakage of exudates, which occurs during the structural destruction of meat during freezing and thawing. The exudate is composed of free amino acids, sugars, and nucleotides, and it can be a good nutrient source for microorganisms^19,20^. The collapse of muscle cell structures during freezing and thawing can result in cavities and meat exudates, which provide optimal conditions for microbial growth (Table 1)^20,21^. According to previous studies that used the minimal spoilage criterion TAB > 6.0 log CFU/g, the shelf life of chicken breast meat was less than 10 and 7 days for unfrozen and FT breast meat, respectively^9^. In unfrozen breast meat, only VBN demonstrated a significant increase along with TAB on day 10, whereas increases in a^*^ value, drip loss, and 2-thiobarbituric acid reactive substances (TBARS) value were not significant when the TAB approached the spoilage threshold (*p* < 0.05). In a previous study, the susceptive difference of VBN was used to monitor the freshness of chicken breast meat at a minimal spoilage level using colorimetric array analysis^5^. However, in FT breast meat, there were no correlation between drip loss and TAB levels and physicochemical properties at the initial spoilage level, respectively (Table 1). A previous study with similar results reported that no direct correlation existed between TBARS and quality attributes^22^. Microorganisms can decrease lipid oxidation compounds in raw and fermented meat products through their own antioxidant activity^23^, and unsaturated aldehydes, which are indicators of lipid peroxidation development, are eliminated during fermentation^24^. Additionally, various microorganisms have their own antioxidative activities, such as generating non-enzymatic antioxidants or reactive oxygen species scavengers to protect themselves from oxidative damage^25^. On the other aspect, owing to low fat contents of chicken breast meat, TBARS did not show a significant difference on the value^2^.

**Table 1:** Total aerobic bacteria and physicochemical properties of unfrozen and frozen/thawed (FT) chicken breast meat during storage.

		Storage (day)							SEM^1^
		0	1	4	7	10	13	16	
Total aerobic bacteria (log CFU/g)	Unfrozen	3.01^ey^	3.05^ey^	3.87^dy^	5.36^cy^	6.06^by^	5.98^by^	6.42^ay^	0.110
	FT	3.87^dx^	4.21^dx^	5.13^cx^	6.57^bx^	7.05^ax^	7.38^ax^	7.18^ax^	0.136
SEM^2^		0.098	0.108	0.094	0.114	0.209	0.095	0.096	
Drip loss (%)	Unfrozen	—	0.74^by^	0.94^by^	1.29^by^	1.85^by^	2.49^ab^	3.80^a^	0.482
	FT	1.00^b3^	2.99^ax^	2.51^ax^	2.84^ax^	3.14^ax^	3.08^a^	3.79^a^	0.448
SEM^2^			0.408	0.335	0.148	0.382	0.444	0.829	
L* (Lightness)	Unfrozen	53.81 ^y^	54.25	53.55	54.15	53.46	54.52	55.28	0.777
	FT	57.18^x^	55.78	56.28	54.45	55.86	55.91	55.32	1.223
SEM^2^		0.521	1.559	1.028	1.010	0.763	0.734	1.203	
a* (Redness)	Unfrozen	4.44^by^	5.03^by^	5.66^aby^	5.65^aby^	5.87^aby^	5.79^aby^	6.69^ay^	0.350
	FT	6.30^bx^	6.87^abx^	7.25^abx^	8.64^ax^	8.53^ax^	8.07^ax^	7.75^abx^	0.421
SEM^2^		0.426	0.309	0.418	0.414	0.415	0.418	0.281	
b* (Yellowness)	Unfrozen	13.35 ^y^	14.69 ^y^	13.43 ^y^	13.31 ^y^	13.60 ^y^	14.05 ^y^	13.51 ^y^	0.588
	FT	17.58^x^	17.81^x^	17.67^x^	16.29^x^	18.23^x^	16.02^x^	15.77^x^	0.758
SEM^2^		0.725	0.759	0.656	0.667	0.788	0.572	0.545	
pH	Unfrozen	6.16^x^	6.17	6.20^x^	6.08	6.17	6.16	6.07	0.066
	FT	5.95 ^y^	6.18	6.01 ^y^	6.14	6.17	6.16	6.16	0.062
SEM^2^		0.048	0.092	0.567	0.039	0.066	0.056	0.076	
TBARS (mg malondialdehyde/kg)	Unfrozen	0.30^b^	0.30^b^	0.28^b^	0.30^b^	0.32^b^	0.34^ab^	0.38^a^	0.016
	FT	0.29	0.33	0.31	0.30	0.31	0.36	0.31	0.021
SEM^2^		0.015	0.017	0.018	0.013	0.013	0.027	0.023	
VBN (mg/100 g)	Unfrozen	10.10^c^	10.55^c^	10.78^c^	11.34^cy^	13.59^b^	17.02^a^	17.89^ay^	0.753
	FT	10.48^d^	11.06^d^	11.12^d^	12.75^dx^	15.75^c^	18.77^b^	22.22^ax^	0.658
SEM^2^		0.230	0.343	0.448	0.280	0.801	0.917	1.252	

^1^Standard of the mean (*n* = 35).

^2^(*n* = 10).

^3^The value represents thawing loss (%) on day 0.

^a–e^Different letters in the same group indicate a significant difference (*p* < 0.05).

^x–y^Different letters in the same storage day indicate a significant difference (*p* < 0.05).

Of the CIE values, only the a* value significantly increased in a time-dependent manner. The L* value was not different in either treatment group, except on the initial storage day. Regardless of storage day, FT breast meat showed high b* value than unfrozen breast meat. According to previous study, color a* value only affected in a time dependent manner, whereas b* value only affected by freezing methods such as freezing rate and air composition^26^. There was no significant difference in pH between unfrozen and FT breast meat on different storage days. Likewise, a previous study^27^ showed that pH of approximately 6.0, which is similar to that used in the present study, was suitable for microbial growth. According to Rukchon et al.^9^, the initial spoilage level of skinless chicken breast was 6.0 log CFU/g. Based on the results, only changes of VBN are closely related to initial spoilage level, demonstrating that VBN can be used as a freshness indicator of chicken breast meat in both unfrozen and FT breast meat. In addition, some relationships are still unclear. Further experiments are needed to investigate this relationship between unfrozen and FT breast meat. It would have been more helpful if we knew the initial state of the FT.

### Metabolites

**Fig. 1:**
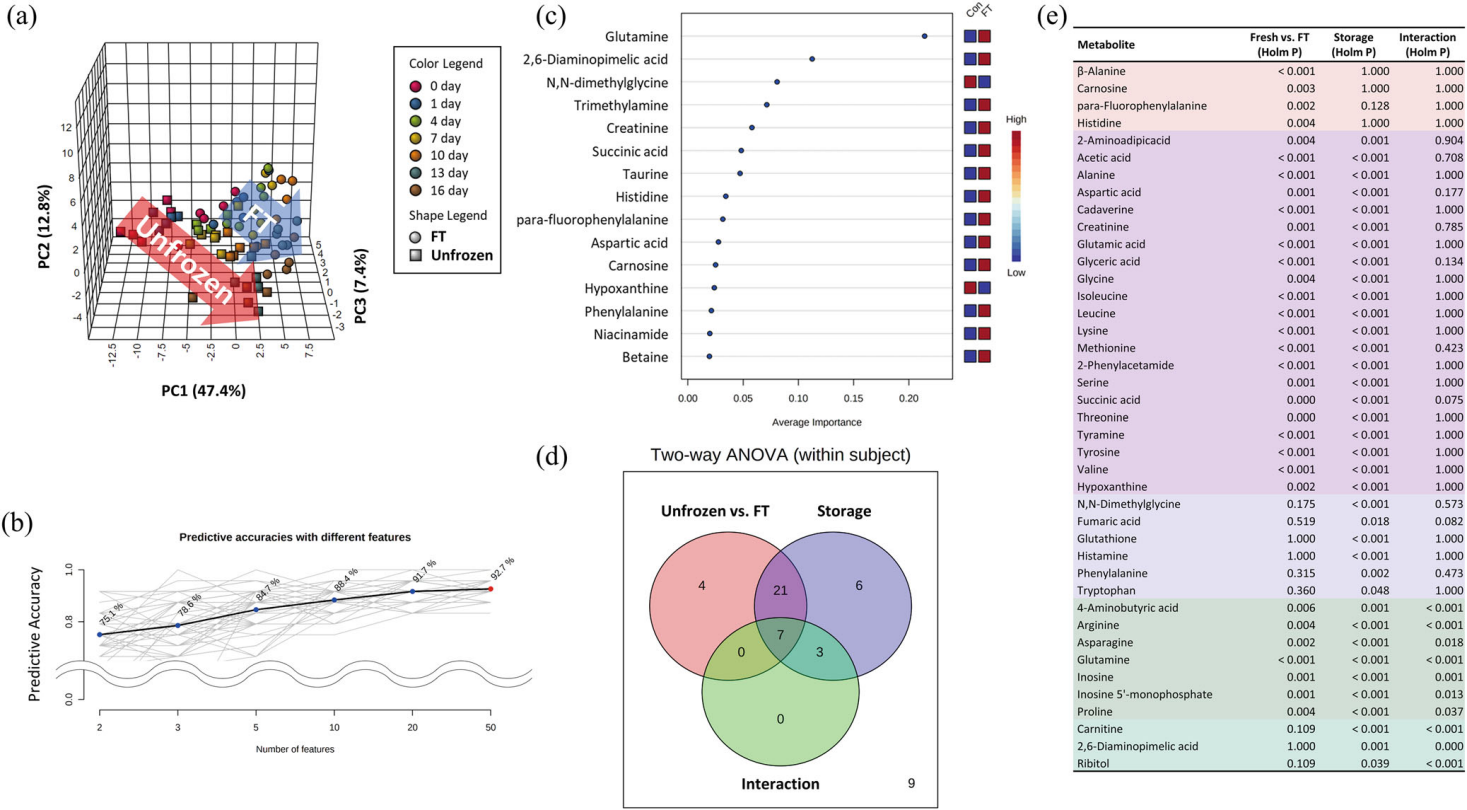
Trends in whole metabolic changes of unfrozen and frozen/thawed chicken meat by storage time. Interactive principal component analysis (iPCA) (**a**), predictive accuracies with different features (**b**), average importance with 50 features prediction model (**c**), Venn diagram of two-way ANOVA (**d**), and list of metabolites which have significance (**e**).

**Fig. 2:**
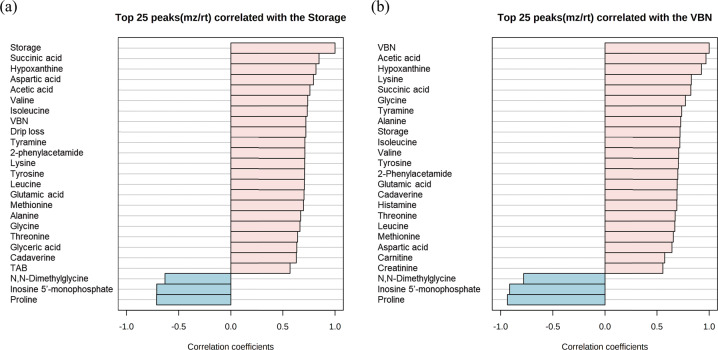
Correlation of physicochemical properties and metabolites. Top 25 items correlated by storage day (**a**) and volatile basic nitrogen (VBN) (mg%) (**b**).

For the evaluation of freshness indicators in chicken breast meat, 2D ^1^H-^13^C HSQC NMR spectra were acquired and processed by multivariate analysis (Fig. 1; Supplementary Table 2). The correlation analysis was performed to determine common trends in both unfrozen and FT breast meat in a time-dependent manner (Fig. 2). The two-way analysis of variance (ANOVA) results (Fig. 1d, e) demonstrated that the quantities of most metabolites were increased gradually to the storage time. The quantities of organic acid, hypoxanthine, and free amino acids were highly correlated with storage time, whereas those of proline, inosine 5´-monophosphate (IMP), and N,N-dimethylglycine (DMG) demonstrated highly negative correlations (Fig. 2). These results indicate that metabolic changes can be used as evidence of microbial growth. Succinate and acetate were representative organic acids produced by microorganisms during microbial fermentation^28^. Increases in acetic acid and succinic acid can result from glucose fermentation by microorganisms, and this change can be observed in muscle-based foods during storage^28–30^. According to Bórquez et al.^31^, acetic acid can suppress microbial counts in fish flesh, but the microorganisms steadily grow in a time-dependent manner, even in 5 mg/kg acetic acid.

An increase in hypoxanthine with a decrease in IMP during storage is a typical change that allows prediction of the freshness of chicken meat using *K*-value (%)^6^. In addition to changes in nucleotides, free amino acids (aspartic acid, valine, isoleucine, lysine, tyrosine, leucine, glutamic acid, methionine, alanine, glycine, and threonine), and biogenic amines (tyramine and cadaverine), there can be evidence of proteolysis by both meat enzymes and microbial activity during storage^10,21^. Increases in VBN and biogenic amines are normally accompanied by food spoilage and are treated as indicators of freshness in meat^5,10,32^. In addition, DMG and proline can be consumed by microorganisms as carbon or nitrogen sources, and a decrease in DMG and proline is evidence of microbial growth^11,12,33^. As indicated above and in agreement with previous studies, DMG and proline levels decreased during storage (*p* < 0.05). VBN levels were more strongly correlated with metabolites (especially acetic acid, hypoxanthine, lysine, and succinic acid) than that with TAB (Fig. 2a, b). This may be a result of stagnating microbial growth in the later storage stage (after minimal spoilage TAB level); TAB did not correlate with VBN and acetic acid at different storage times (Fig. 2a). Considering the physicochemical properties of chicken breast meat, acetic acid had a strong correlation with VBN (Pearson *r* = 0.97), and could act as an appropriate freshness indicator (Fig. 2b).

### Unfrozen and FT breast meat

#### Overall metabolic trends by multivariate analysis

To delineate and identify the relationships among metabolite changes, storage time, and freshness, Interactive principal component analysis (iPCA), ROC analysis, and two-way ANOVA were performed (Fig. 1). iPCA results revealed a strong correlation between PC1 (47.4%) and storage time. In addition to PC1, the use of the PC1–PC2 (12.8%) plane explained the difference better by treatment (unfrozen and FT breast meat) and storage time than the use of PC1 only (Fig. 1a; Supplementary Table 1). Unfrozen breast meat was clearly distinguished from the other clusters until day 1. From day 4, the clusters of unfrozen breast meat moved in a similar direction to FT breast meat over time but did not overlap. The clusters of FT breast meat moved along PC1 and PC2 over time. In PC3 (7.4%), the unfrozen breast meat cluster had a negative correlation, whereas the FT breast meat cluster was positively correlated over time. PC3 supports the explanation of overall variation, but it cannot be used independently. Prior to selecting metabolic indicators for distinguishing both groups, receiver operating characteristic (ROC) curve analysis and two-way ANOVA were used for the selection of indicators (Fig. 1b, c). The highest predictive accuracy was the 92.7% for the model made with 50 features. Among them, a total of 41 metabolites that were significantly different were placed on the Venn diagram (*p* < 0.05) (Fig. 1d, e). For the selection of indicators, we used a two-way ANOVA. In the Venn diagram, four metabolites were significant only in unfrozen vs. FT breast meat, and interactions were chosen as candidates for ROC curve analysis because the top 15 metabolites with average importance among the 50 features were not directly related to the results of the two-way ANOVA.

#### Enhancing discriminant ability with selected metabolites

**Fig. 3:**
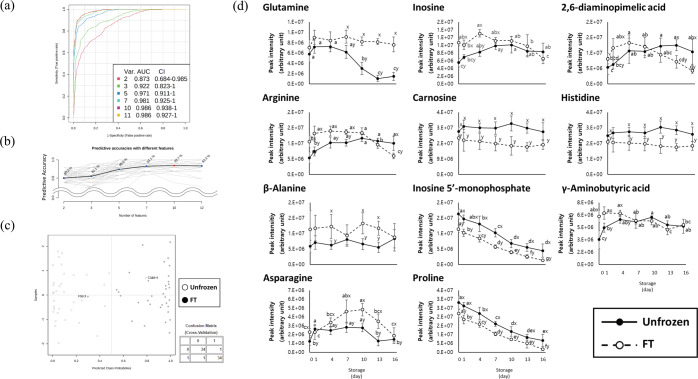
Key metabolites for discrimination of unfrozen and frozen/thawed chicken meat. Multivariate area under the curve (AUC) analysis (**a**), predictive accuracies with different features (**b**), predicted class probabilities (**c**), and changes over storage time (**d**) made with 11 selected metabolites from two-way analysis of variance (ANOVA). ^a–f^Different letters in the same group indicate a significant difference (*p* < 0.05). ^x–y^Different lettersin the same storage day indicate a significant difference (*p* < 0.05).

Among the various combinations of candidates, 11 metabolites were selected based on their prediction score, and ROC analysis was performed (Fig. 3). The ROC curves consisting of the selected metabolites had higher predictive accuracy than that of the original model using the whole metabolome, and the accuracy was highest when 10 features were used (Fig. 1b; Fig 3a–c). The metabolites are listed in order of mean importance from most to least important in Fig. 3d. Quantifying the metabolites is important for evaluating meat quality such as flavor-active and bioactive compounds^34^. Furthermore, this information can also be used for distinguishing between groups using a machine-learning algorithm^18^. The quantity of glutamine rapidly decreased after day 10 in unfrozen breast meat (0.48-fold) but remained constant throughout the storage period in FT breast meat (*p* < 0.05). In contrast, asparagine gradually increased in unfrozen breast meat but decreased after day 13 in FT breast meat. Asparagine demonstrated a similar trend in both groups, but it was higher in FT breast meat during initial and terminal storage than in unfrozen breast meat. Proline content decreased gradually in both groups but it was higher in unfrozen breast meat, at 1.28-fold change on day 0 followed by a 3.81-fold change by day 16, than in FT breast meat (*p* < 0.05). Glutamine, arginine, asparagine, and proline can be utilized by microorganisms, and the utilization of free amino acids varies depending on microbial species^12,35–37^. However, some free amino acids demonstrated different behavior in unfrozen meat compared with that in FT meat. It can be inferred that freezing and thawing could change the microbial composition through the competitive exclusion of microorganisms, resulting in the survival of cold-resistant species and altering the metabolite consumption trend^38^.

Previous studies reported that 2,6-diaminopimelic acid (DAPA) and γ-aminobutyric acid (GABA) are direct evidence of microbial proliferation^12,39^. DAPA is a component of cell wall peptidoglycan in various bacteria and is a precursor of lysine^40^. The presence of DAPA can be used to evaluate microbial contamination and the existence of lactobacilli^41,42^. Based on these results, FT breast meat is more vulnerable to microbial spoilage than unfrozen breast meat because of the exudates during thawing. They contain suitable nutrients for microbial growth, resulting in a high TAB and influencing metabolic changes (an increase in DAPA and GABA and a decrease in proline) in meat. IMP was gradually decreased and could be used to clearly distinguish between unfrozen and FT breast meat, whereas inosine could be used to distinguish between unfrozen and FT breast meat only before day 10. In addition to being freshness indicators, higher carnosine and IMP content can enhance the umami flavor of chicken breast meat^43^. Carnosine (β-alanyl-L-histidine) and anserine (β-alanyl-3-methyl-L-histidine) are histidine dipeptides composed of β-alanine and histidine, which have various bioactivities such as antioxidant activity, a buffering effect, and the potential for metal ion chelation and free radical scavenging. They can also act against various diseases^10^.

In the present study, carnosine in FT breast meat appeared to decompose into β-alanine and histidine during freezing and thawing. A similar trend was observed in a previous study, in which the authors suggested that the decrease in carnosine might be the result of degradation by oxidative stress during freezing and thawing^44^. However, carnosine is typically transformed by various oxidants (free radicals and lipid peroxidation byproducts) and degraded to various derivatives, instead of being degraded to β-alanine and histidine^45^. The decrease in carnosine content during freezing and thawing has rarely been reported in chicken meat. The concentrations of carnosine, histidine, and β-alanine were relatively consistent throughout the storage period. Contrasting results have also been reported for increasing or consistent trends in carnosine levels during storage, and this difference might result from carnosinase activity levels^46^. Based on these results, proteolysis and microbial changes were not relevant to changes in carnosine levels. To understand the decrease in carnosine levels during freezing and thawing, further analysis is needed.

**Fig. 4:**
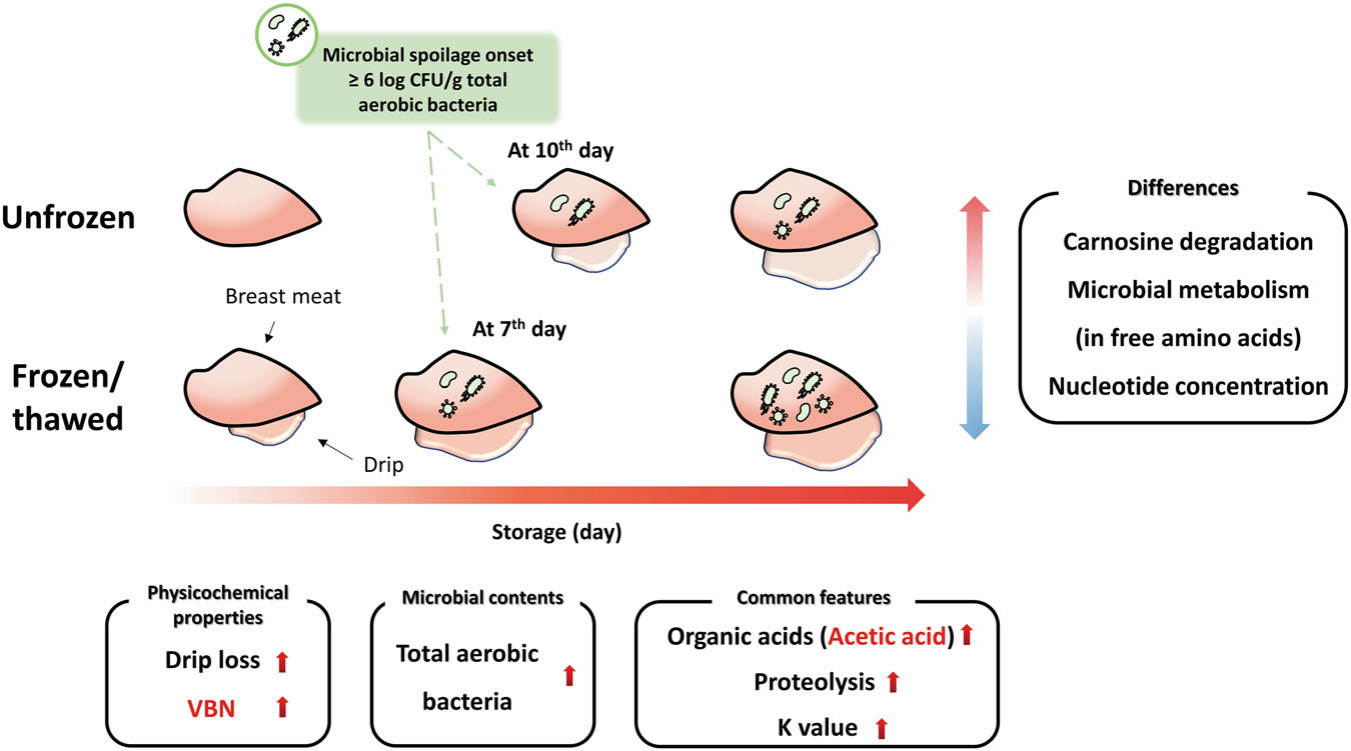
Schematic representation of overall physicochemical and metabolic changes in unfrozen and frozen/thawed chicken breast meat. Red colored letters represent the most suitable candidates for identifying chicken freshness based on the microbial spoilage level.

A summarized schematic illustration of the present study is represented in Fig. 4. Among all physicochemical properties, only VBN could predict the spoilage of chicken breast meat at the initial TAB level. Generally, proportional increases in organic acids, free amino acids, biogenic amines, and hypoxanthine were observed, while proline and DMG decreased proportionally during storage. Acetic acid was highly correlated with VBN, which could be a simple indicator of freshness in chicken breast meat without VBN analysis for both unfrozen and FT breast meat. Unfrozen and FT breast meat can be differentiated by uniform concentration of carnosine, β-alanine, and histidine levels, consistent changes in nucleotides by storage time, and changes in microbial metabolism patterns that are reflected by some free amino acids. Based on these results, 2D NMR-based metabolomics could be useful for evaluating the freshness of chicken breast meat. In addition, it can be used to differentiate unfrozen from FT breast meat based on different metabolic patterns.

## Methods

### Sample preparation

Five packages (1 kg/package) for unfrozen and frozen experiment groups of skinless chicken (cobb 500 f) breast meat were purchased on the same day from same slaughterhouse (Harim Co. Ltd. Iksan, South Korea), respectively. All chickens were slaughtered in an automated commercial slaughterhouse system. Carbon dioxide stunning and air-chilling (carcasses were chilled to 2 °C and the temperature was kept at about 3.5 h during rigor mortis) were used. For preparation of FT breast meat, each breast meat was rapidly frozen at −35 °C immediately after slaughter, with the aim of reaching −20 °C in 40 min, packed randomly, and subsequently stored at −20 °C. Breast meat (5 packages/group) was packaged by polystyrene and styrofoam tray, transferred to the laboratory using a cooler with ice. It took about 3 h from chicken processing plant to the laboratory. For simultaneous analysis with unfrozen breast meat, frozen breast meat was thawed 24 h before the unfrozen breast meat samples were collected, and both samples were prepared at the same facility. From the same package (8–9 breasts/package), seven breasts were randomly selected, and these were packaged aerobically and individually in low-density polyethylene/nylon bags (oxygen permeability of 4.7 g/m^2^ for 24 h at 100% RH/25 °C) and marked with the same number. The breast meat was stored for up to 16 days (0, 1, 4, 7, 10, 13, and 16) at 2 °C. After drip loss and color were evaluated daily in quintuplicate, each chicken breast meat was minced using a mini chopper (CH180, Kenwood Appliances Co., Ltd., Dongguan, China). TAB and pH analyses were performed immediately. Samples for TBARS, VBN, and NMR analyses were weighed, vacuum-packaged, and stored at −70 °C until further analyses.

### TAB counts

TAB counts were performed according to the method described by Yong et al.^47^. Each sample (3 g) was diluted in 27 mL of sterile saline (0.85%) for 2 min using a stomacher (BagMixer^®^ 400 P, Interscience Ind., St. Nom, France). Appropriate dilutions were prepared in sterile saline and spread on plate count agar (Difco Laboratories, Franklin Lakes, NJ, USA). The agar plates were incubated at 37 °C for 48 h, and the microbial counts were calculated. The results are expressed as log numbers of colony-forming units per gram (log CFU/g).

### Drip loss

The breast meat was weighed before packaging. Each day during storage, drips were collected to evaluate drip loss. At the first day, in case of FT breast meat, thawing loss was evaluated using each whole pack instead of drip loss and each FT breast meat was packaged aerobically for further storage. Drip loss was represented as a percentage and calculated as follows:

$${{{\mathrm{Drip}}}}\,{{{\mathrm{loss}}}}\left( {{{\mathrm{\% }}}} \right) = \frac{{original\,sample\,weight - sample\,weight\,after\,storage}}{{original\,sample\,weight}} \times 100$$

### Color

Surface color measurements (CIE L*, a*, and b* values representing lightness, redness, and yellowness, respectively) of chicken breast meat samples (skin side of *Pectoralis major*) were obtained using a colorimeter (CM-5, Konica Minolta Censing Inc., Osaka, Japan) in the condition of illuminant D65 and 10° standard observer with a 30 mm aperture size plate. Calibration was performed using a calibration plate. The color of each sample was measured three times along the skin side to minimize error and the average value was used.

### pH

Chicken breast meat (1 g) was homogenized with 9 mL of distilled water using a homogenizer (T25 Ultra, Ika Works, Staufen, Germany). The homogenates were centrifuged (Continent 512 R, Hanil Co., Ltd., Gimpo, South Korea) at 2,265 × *g* for 10 min and filtered (Whatman No. 4, Whatman PLC, Middlesex, UK). The pH value of each filtrate was measured using a pH meter (Seven2Go S2, Mettler-Toledo International Inc., Schwerzenbach, Switzerland). Prior to measuring the pH, the pH meter was calibrated using standard buffers (pH 4.01, 7.00, and 9.21).

### TBARS

Chicken breast meat (5 g) was homogenized with 15 mL of distilled and deionized water (DDW) and 50 µL of butylated hydroxyl toluene (in ethanol) for 30 s at 1513 × *g*. The homogenate (2 mL) was transferred to a 15 mL test tube and mixed with 4 mL of TBA and trichloroacetic acid (20 mM TBA in 15% trichloroacetic acid). The test tubes were heated in a water bath at 90 °C for 30 min, cooled, and centrifuged (Continent 512 R, Hanil Co., Ltd.) at 2265 × *g* for 10 min. The absorbance of the supernatant was measured at 532 nm using a spectrophotometer (X-ma 3100, Human Co. Ltd., Gwangju, South Korea). The TBARS value was calculated as mg malondialdehyde/kg sample.

### VBN

The VBN content was evaluated using the Conway micro-diffusion technique with slight modifications^48^. Chicken breast meat (5 g) was homogenized with 20 mL of DDW for 30 s at 1,130 × *g*. The homogenate was filtered using a filter paper (Whatman No.1, Whatman PLC, Middlesex, UK). Further, 1 mL of filtrate and K_2_CO_3_ were placed at both end sides separately into the outer space of the Conway tool (Sibata Ltd., Saitama, Japan), and 1 mL of 0.01 N H_3_BO_3_ and 100 μL of Conway reagent (0.066% methyl red:0.066% bromocresol green, 1:1) were added to the inner space, and the Conway tool was sealed with grease.

1 $${{{\mathrm{VBN}}}}\,{{{\mathrm{mg\% }}}}({{{\mathrm{mg}}}}/100\,{{{\mathrm{g}}}}\,{{{\mathrm{sample}}}}) = 0.14 \times ({{{\mathrm{a}}}} - {{{\mathrm{b}}}}) \times 5 \times 100$$

where *a* is the titration volume of 0.01 N HCl (mL) in the sample and *b* is the titration volume of 0.01 N HCl (mL) in the blank.

### Preparation of chicken breast meat for NMR analysis

Chicken breast meat (5 g) was thawed at 4 °C for 30 min. Thawed chicken breast meat was homogenized at 1,720 × *g* for 30 s (T25 basic, Ika Co., Kg, Staufen, Germany) with 20 mL of 0.6 M perchloric acid. The homogenate was centrifuged (Continent 512 R, Hanil Co., Ltd., Incheon, South Korea) at 3,086 × *g* for 15 min at 4 °C. Each supernatant was transferred to a new test tube and neutralized with KOH. The neutralized extracts were centrifuged again under the same conditions. After centrifugation, the supernatant was filtered using a filter paper (Whatman No. 1, Whatman PLC) and lyophilized (Freezer dryer 18, Labco Corp., Kansas City, MO, USA). The lyophilized extracts were stored at −70 °C until NMR analysis. Prior to NMR analysis, lyophilized extracts were reconstituted by 1 mL of 1 mM 3-(trimethylsilyl)propionic-2, 2,3, 3-*d*_*4*_ acid (TSP) deuterium oxide solution [20 mM phosphate buffer system titrated with pD 7.0]. TSP was used for the purpose of aligning the NMR spectrum to 0.00 ppm. Reconstituted extracts were kept at 35 °C for 10 min to dissolve metabolites well and then centrifuged at 3,086 × *g* for 15 min at 4 °C. The 800 μL of supernatant was transferred to microtube and centrifuged at 17,000 × *g* for 15 for 10 min (HM-150IV, Hanil Co., Ltd.). The 600 μL of final supernatant were transferred to 5 mm NMR tube for NMR analysis.

### NMR analysis

All NMR spectra were recorded in D_2_O at 298 K using a Bruker 850 MHz Cryo-NMR spectrometer (Bruker Biospin GmbH, Rheinstetten, Baden-Württemberg, Germany). 1D ^1^H NMR was performed by applying standard zg30 with slight modification (recycle delay of 1 s) default in Topspin 3.6.2 (Bruker Biospin GmbH). The 1D ^1^H NMR experiment was performed using 64 k data points, a sweep width of 17,007.803 Hz, and 128 scans. According to Kim et al.^17^, two-dimensional NMR analyses [^1^H-^1^H correlation spectroscopy (COSY)^1^,H-^1^H total correlation spectroscopy (TOCSY)^1^,H-^13^C heteronuclear single quantum coherence (HSQC), and ^1^H-^13^C heteronuclear multiple-bond correlation spectroscopy (HMBC)] were performed to quantify and qualify the metabolites (Supplementary Table 2). COSY and TOCSY experiments were performed with 2 k data points in the t_2_ domain and 256 increments in t_1_, each with 8 and 16 scans, respectively. Spectral widths of 11 ppm were used for TOCSY experiments. HSQC and HMBC experiments were performed with 2 k data points in the t_2_ domain and 512 increments in t_1_, each with 8 and 32 scans, respectively. The spectral widths were 11 ppm for the f_2_ dimension and 180 and 240 ppm for the f_1_ dimension. Coupling constant values of 145 and 8 Hz were employed to set the delay durations for short-range and long-range correlations, respectively. To quantify the metabolites, acquired HSQC spectra were processed using Analysis of MIXtures software v3.9 (Bruker Biospin GmbH). Among assigned peaks of each metabolite (Supplementary Table 1), non-overlapped peak with good intensity was selected as candidate. To quantification of peak, all HSQC spectra were loaded and overlapped on Analysis of MIXtures software. Selected peak ranges were set manually for automation process, then all spectra were quantified at once^10,17^. After quantification, we reviewed the dataset and organized it according to the format for further analysis.

### Statistical analysis

The acquired integrated HSQC data was used for multivariate analysis. Multivariate analysis was performed using MetaboAnalyst 4.0 (www.metaboanalyst.ca). Prior to multivariate analysis, integrated data were filtered interquartile range to eliminate outlier, log-transformed without normalization and mean-centered and divided by the standard deviation of each variable for all multivariate analyses. iPCA and two-way ANOVA were performed to distinguish between unfrozen and FT meat based on treatment and storage time. Additionally, quantified metabolites, physicochemical properties, and TAB were combined for correlation analysis using pattern hunter analysis. Discriminant analysis was completed using ROC curve analysis with linear support vector machine (SVM) classification. The feature metabolites were ranked based on the SVM’s built-in ranking method. Statistical analysis for selected metabolites in the figure of discriminant analysis was performed using the procedure of the general linear model for the comparison of quantified metabolites from HSQC. Significant differences were determined using the Student–Newman–Keuls multiple range test with the SAS software (SAS 9.4, SAS Institute, Cary, NC, USA) at a confidence level of *p* < 0.05. All experiments were conducted in quintuplicate. TAB counts, color, TBARS, and VBN were conducted with two or three observations per replication and the values were averaged for statistical analysis.

## Supplementary information

Supplementary Material

The results for Split-Plot Design

## Data Availability

The authors declare that all relevant data supporting this study has been included within the paper. Raw data will be made available by corresponding authors upon reasonable request.
