# Supplementary material for: Using 2D qNMR analysis to distinguish between frozen and frozen/thawed chicken meat and evaluate freshness
Source: NPJ Sci Food. 2022 Sep 22;6:44. doi: 10.1038/s41538-022-00159-x (PMC9500024; doi:10.1038/s41538-022-00159-x)
Supplement: Supplementary file 1 — Supplementary Material [file 41538_2022_159_MOESM1_ESM.pdf]

Supplementary Table 1. Loadings matrix for the interactive principal component analysis (iPCA) of Figure 1(a).

Variables	Loadings		
	PC1	PC2	PC3
Anserine	-0.999	0.011	0.118
N,N-dimethylglycine	-0.834	0.300	-0.030
Inosine 5'-monophosphate	-0.164	0.206	-0.053
Proline	-0.157	0.233	-0.060
para-fluorophenylalanine	-0.146	-0.063	-0.186
Carnosine	-0.127	-0.088	-0.318
Histidine	-0.108	-0.096	-0.335
Lactate	-0.082	0.045	-0.208
Glutathione	-0.080	-0.178	0.118
Adenosine 5'-diphosphate	-0.079	0.074	0.178
Creatine	-0.024	-0.047	-0.268
Glutamine	0.000	0.212	0.267
myo-Inositol	0.013	0.187	0.062
Trimethylamine	0.022	0.093	0.198
Ribitol	0.030	0.200	-0.280
Betaine	0.035	0.047	0.152
Taurine	0.059	0.015	0.036
Inosine	0.071	0.269	-0.210
Histamine	0.080	-0.261	-0.051
Niacinamide	0.081	0.194	-0.149
D,L-2,6-Diaminopimelic acid	0.084	0.196	-0.168
4-Aminobutyric acid	0.091	0.222	-0.156
Tryptophan	0.094	0.200	-0.023
Asparagine	0.094	0.212	0.135
Fumarate	0.100	-0.093	-0.143
Phenylalanine	0.111	0.044	0.055
beta-Alanine	0.117	0.125	0.216
Arginine	0.122	0.247	-0.153
Carnitine	0.135	-0.063	-0.168
Creatinine	0.149	-0.053	-0.190
2-Aminoadipic acid	0.149	0.149	-0.005
Serine	0.150	0.216	-0.055
Acetic acid	0.160	-0.236	0.018
Glyceric acid	0.161	0.049	-0.093
hypoxanthine	0.179	-0.168	0.013
Aspartic acid	0.185	0.009	-0.862
Succinate	0.189	-0.095	-0.093
Glycine	0.190	-0.055	0.066
Cadaverine	0.193	-0.001	0.063
Threonine	0.195	0.017	0.080
L-lysine	0.196	-0.081	0.049
Alanine	0.197	-0.014	0.068
Glutamic acid	0.197	-0.003	-0.010
Methionine	0.202	0.022	-0.050
Tyramine	0.203	-0.008	0.012
Leucine	0.203	0.023	-0.027
Valine	0.203	0.000	-0.020
Tyrosine	0.204	0.009	-0.002
2-phenylacetamide	0.204	0.006	-0.021
Isoleucine	0.204	-0.002	-0.010

Supplementary Table 2. Signal assignment of NMR experiments (^{1}H, COSY, TOCSY, HSQC, and HMBC) from chicken breast meat.

Peak	Compound	Abbreviation	Group	^{1}H(ppm)	Mult.: J^{1} (Hz)	Assignment data
1	Isoleucine	Ile	δ-CH_3	0.95	*t*: 7.52	COSY(1.28, 1.49) TOCSY(2.10) HSQC(14.1) HMBC(19.5, 27.3, 38.7)
2	Leucine	Leu	δ-CH_3/ δ-CH_3	0.97/0.98	*dd*: 6.13/6.12	COSY(1.74) TOCSY(3.76) HSQC(23.8/24.9) HMBC(27.0, 42.6)
3	Valine	Val	γ-CH_3	1.01	*d*: 7.06	COSY(2.30) TOCSY(3.64) HSQC(19.5) HMBC(20.77, 32.0, 63.1)
4	Isoleucine	Ile	δ-CH_3	1.03	*d*: 7.06	COSY(2.01) TOCSY(1.27;1.47;3.68) HSQC(17.40) HMBC(27.3, 38.7)
5	Valine	Val	γ-CH_3	1.06	*d*: 7.06	COSY(2.30) TOCSY(3.64) HSQC(20.77) HMBC(32.0, 63.1)
6	Isoleucine	Ile	γ-CH_2	1.28	*m*	COSY(0.95;1.49) TOCSY(2.10) HSQC(27.3) HMBC(14.1, 38.7)
7	Threonine	Thr	γ-CH_3	1.36		COSY(4.29) TOCSY(3.63)
8	Lactic acid	LA	CH_3	1.36	*d*: 6.97	COSY(4.15) HSQC(23.0) HMBC(71.4)
9	Lysine	Lys	γ-CH_2	1.48	*m*	COSY(1.74) TOCSY(3.04) HSQC(25.0)
10	Isoleucine	Ile	γ'-CH_2	1.49	*m*	COSY(0.95, 1.28) TOCSY(2.10) HSQC(27.3) HMBC(14.1, 38.7)
11	Cadaverine		γ-CH_2	1.49	*m*	COSY(1.74) TOCSY (3.04) HSQC (25.5) HMDB (29.2, 42.5)
12	Alanine	Ala	β-CH_3	1.51	*d*: 7.31	COSY(3.81) HSQC(19.0) HMBC(53.3, 178.7)
13	Leucine	Leu	β-CH_2	1.71	*m*	COSY(0.97, 0.98, 3.74) HSQC(42.6) HMBC(23.8,24.9)
14	Lysine	Lys	δ-CH_2	1.72	*m*	COSY(3.01) HSQC(29.1)
15	Cadaverine		β,δ-CH_2	1.74	*m*	COSY (3.04) HSQC (29.2) HMDB (25.5, 42.5)
16	Leucine	Leu	β'-CH_2	1.75	*m*	COSY(0.97, 0.98, 3.74) HSQC(42.6) HMBC(23.8,24.9)
17	Lysine	Lys	β-CH_2	1.94	*m*	COSY(1.48, 3.78) TOCSY(1.72, 3.04) HSQC(32.7) HMBC(29.1, 57.2)
18	Acetic acid		CH_3	1.94	*s*	HSQC(26.2)
19	Isoleucine	Ile	β-CH	2.00	*m*	COSY(1.03) TOCSY(0.95) HSQC(38.7)
20	Glutamate	Glu	β-CH_2	2.09	*m*	COSY(2.16, 2.38) HSQC (29.8) HMBC(36.2, 57.4, 177.4, 184.1)
21	Methionine	Met	S-CH_3	2.15	*s*	HSQC(16.6) HMBC(31.6)
22	Aspartic acid	Asp	β-CH_2	2.15	*m*	COSY(2.22, 2.66) HSQC(32.6)
23	Glutamate	Glu	β'-CH_2	2.16	*m*	COSY(2.09, 2.38) HSQC(29.8) HMBC(36.2, 57.4, 177.4, 184.1)
24	Glutamine	Gln	β-CH_2	2.16	*m*	COSY(2.47, 3.80) HSQC(29.1) HMBC(33.6, 56.9, 177.0, 180.4)

25	Glutathione	GHS	β,β'-CH_2	2.17	*m*	COSY(2.66, 3.80) HSQC(29.1)
26	Aspartic acid	Asp	β'-CH_2	2.22	*m*	COSY(2.15, 2.66) HSQC(32.6)
27	4-Aminobutyric acid	GABA		2.25		HSQC(36.4)
28	Valine	Val	β-CH	2.3	*m*	COSY(1.01, 1.06) HSQC(32.0) HMBC(19.5, 176.9)
29	Glutamate	Glu	γ-CH_2	2.37	*m*	COSY(2.09, 2.16) HSQC (36.2) HMBC(29.8, 57.4, 177.4, 184.1)
30	Succinic acid		α,β-CH_2	2.43	*s*	HSQC(36.9)
31	Glutamine	Gln	γ-CH_2	2.45	*m*	COSY(2.16) TOCSY (3.80) HSQC(33.6) HMBC(29.1, 56.9, 177.0, 180.4)
32	carnitine	Cart	α-CH_2	2.46		COSY(4.59) TOCSY (3.45) HSQC(45.9)
33	Glutamine	Gln	γ'-CH_2	2.5	*m*	COSY(2.16) TOCSY (3.80) HSQC(33.6) HMBC(29.1, 56.9, 177.0, 180.4)
34	β-Alanine	β-Ala	α-CH_2	2.58	*t*: 6.74	COSY(3.21) HSQC(36.5) HMBC(39.3, 181.2)
35	Methionine	Met	γ-CH_2	2.66	*t*: 7.60	HSQC(31.6) HMBC(16.6, 32.6, 56.7, 174.4)
36	Aspartic acid	Asp	α-CH	2.66		COSY(2.15, 2.22) HSQC(31.6)
37	Glutathione	GHS	γ-CH_2	2.66	*m*	COSY(2.16) HSQC(33.7)
38	Carnosine	Car	NH_2-CH_2-CH_2	2.71	*m*	COSY(3.24) HSQC(34.9) HMBC(38.4, 174.5)
39	Aspartic acid	Asp	β-CH_2	2.75	*dd*	COSY(2.83, 3.93) HSQC(39.3) HMBC(54.9)
40	Anserine	Ans	NH_2-CH_2-CH_2	2.75	*m*	COSY(3.24) HSQC(34.9) HMBC(38.4, 174.5)
41	Aspartic acid	Asp	β'-CH_2	2.83	*dd*	COSY(2.75, 3.93) HSQC(39.3) HMBC(54.9)
42	N,N-dimethylglycine	DMG	N-CH_3	2.95	*s*	HSQC (46.3) HMBC (62.7)
43	Lysine	Lys	ε-CH_2	3.03	*m*	COSY(1.72) TOCSY(1.48, 1.94, 3.78) HSQC(41.8)
44	Cadaverine		α,ε-CH_2	3.04	*m*	COSY (3.04) TOCSY (1.49) HSQC (42.5) HMDB (25.5, 29.2)
45	Creatine/Phospho creatine	Cr/PCr	N-CH_3	3.05	*s*	TOCSY(3.94) HSQC(39.8) HMBC(24.3, 151.1, 168.0)
46	Anserine	Ans	β-CH_2	3.08	*m*	COSY(3.25, 4.51) HSQC(28.8) HMBC(56.2, 122.7, 133.4, 174.5, 179.6)
47	Choline			3.15		HSQC(55.0)
48	Carnosine	Car	β-CH_2	3.08	*m*	COSY(3.25, 4.50) HSQC(30.6) HMBC(57.41, 119.8, 133.8, 174.5, 179.9)
49	carnitine	Cart	$N(CH_3)_3$	3.22		HSQC(56.7) HMBC(70.8)
50	Carnosine	Car	β'-CH_2	3.23	*m*	COSY(3.25, 4.50) HSQC(30.6) HMBC(57.41, 119.8, 133.8, 174.5, 179.9)
51	Anserine	Ans	β'-CH_2	3.25	*m*	COSY(3.25, 4.51) HSQC(28.8) HMBC(56.2, 122.7, 133.4, 174.5, 179.6)

52	Anserine	Ans	NH_2-CH_2	3.25	*m*	COSY(3.08, 4.51) HSQC(38.4) HMBC(35.4, 56.2, 122.7, 133.4, 179.6)
53	β-Glucose	β-Glc	CH-2	3.28	*m*	COSY(4.68) HSQC (77.1) HMBC (78.5, 98.8)
54	Betaine	Bet	$N(CH_3)_3$	3.29	*s*	HSQC(56.2) HMBC(69.03)
55	Taurine	Tau	S-CH_2	3.3	*t*	COSY(3.45) HSQC(50.4)
56	myo-Inositol		CH-1,3	3.31	*t*	COSY (3.64, 4.05) HSQC (77.1) HMDB (75.2)
57	β-Glucose	β-Glc	CH-4	3.43	*m*	COSY (3.49) TOCSY(3.74, 4.68) HSQC (72.5) HMBC (63.5, 78.5)
58	α-Glucose	α-Glc	CH-4	3.44	*m*	COSY (3.49) TOCSY(5.26) HSQC (72.5) HMBC (63.5, 78.5)
59	Taurine	Tau	N-CH_2	3.45	*t*	COSY(3.3) HSQC(38.1)
60	carnitine	Cart	γ,γ'-CH_2	3.45	*m*	COSY(4.59) HSQC(72.8)
61	β-Glucose	β-Glc	CH-3	3.49	*m*	TOCSY(4.68) HSQC (78.8)
62	α-Glucose	α-Glc	CH-2	3.56	*m*	COSY(5.26) HSQC (74.2)
63	Glycerol		CH-1	3.57	*m*	COSY(3.66) HSQC(65.5)
64	Glycine	Gly	α-CH	3.59	*s*	HSQC(44.3) HMBC(175.4)
65	myo-Inositol		CH-4	4.05	*t*	COSY (3.31) HSQC (75.2) HMDB (77.1)
66	Threonine	Thr	α-CH	3.63		COSY(4.29) TOCSY(1.36) HSQC(63.20)
67	Valine	Val	α-CH	3.65	*d*	COSY(2.3) TOCSY(1.00;1.05) HSQC(63.1)
68	Glycerol		OH-CH_2	3.66	*m*	COSY(3.57) HSQC(65.3)
69	Isoleucine	Ile	α-CH	3.7	*d*	COSY(2.01) TOCSY(1.03) HSQC(62.3)
70	N,N-dimethylglycine	DMG	α-CH_2	3.75	*s*	HSQC (62.7) HMBC (46.3, 173.3)
71	β-Glucose	β-Glc	CH_2-6	3.75		COSY(3.45; 3.90) HSQC(63.6)
72	α-Glucose	α-Glc	CH-3	3.75		TOCSY(5.26) HSQC(75.5)
73	Leucine	Leu	α-CH	3.76	*m*	COSY(1.71) TOCSY(0.97) HSQC(56.2)
74	Lysine	Lys	α-CH	3.79	*m*	COSY(1.94) TOCSY(3.03) HSQC(57.3) HMBC(32.7)
75	Glutamine	Gln	α-CH	3.8	*m*	HSQC(56.9) HMBC(29.1, 33.6, 177.0)
76	Ribitol		β-CH	3.8	*d*	HSQC(74.8)
77	Anserine	Ans	N-CH_3	3.83	*s*	HSQC(35.4) HMBC(133.4, 138.8)
78	α-Glucose	α-Glc	CH-5	3.86	*m*	TOCSY(5.26) HSQC(74.5)

79	α-Glucose	α-Glc	CH_2-6	3.86	*m*	TOCSY(5.26) HSQC(63.5)
80	Inosine	Ino	CH-5 (Rib)	3.87	*m*	COSY(3.93, 4.29) HSQC(64.2)
81	β-Glucose	β-Glc	CH_2-6'	3.91		HSQC(63.5)
82	Inosine	Ino	CH-5' (Rib)	3.93	*m*	COSY(3.87, 4.29) HSQC(64.2)
83	Betaine	Bet	α-CH_2	3.95	*s*	HSQC(69.03) HMBC(56.2, 172.1)
84	Aspartic acid	Asp	α-CH	3.93	*m*	COSY(2.75, 2.83) HSQC(54.9)
85	Inosine 5'phosphate	IMP	CH_2 (Rib)	4.06/4.09	*m*	COSY(4.40) TOCSY(4.53, 4.79, 6.14) HSQC(66.5)
86	Lactic acid	LA	α-CH	4.15	*q*: 6.94	COSY(1.36) HSQC(71.4) HMBC(23.0)
87	Threonine	Thr	β-CH	4.29		COSY(1.36, 3.63) HSQC(68.8)
88	Inosine	Ino	CH-4 (Rib)	4.29		COSY(3.87, 4.46) TOCSY(3.93, 4.77) HSQC(88.5)
89	Inosine 5'phosphate	IMP	CH-4 (Rib)	4.4	*m*	COSY(4.06, 4.09, 4.53) TOCSY(4.79, 6.14) HSQC(87.6) HMBC(73.4)
90	Inosine	Ino	CH-3 (Rib)	4.46	*dd*	COSY(4.30, 4.77) TOCSY(3.87, 3.93) HSQC(73.2)
91	Carnosine	Car	CH-COOH	4.49	*m*	COSY(3.08, 3.23) HSQC(57.5)
92	Anserine	Ans	CH-COOH	4.51	*m*	COSY(3.08, 3.25) HSQC(56.3)
93	Inosine 5'phosphate	IMP	CH-3 (Rib)	4.53	*dd*:4.72, 4.21	COSY(4.40, 4.79) TOCSY(4.06, 4.09, 6.14) HSQC(73.4) HMBC(66.4, 90.2)
94	carnitine	Cart	β-CH	4.59		COSY(2.45,3.45) HSQC(73.2)
95	β-Glucose	β-Glc	CH-1	4.68	*d*:7.96	COSY(3.28) TOCSY(3.49) HSQC(98.8)
96	Inosine	Ino	CH-2 (Rib)	4.77		COSY(4.46, 6.09) TOCSY(4.29) HSQC(76.9)
97	Inosine 5'phosphate	IMP	CH-2 (Rib)	4.79	*t*: 503	COSY(4.53, 6.14) TOCSY(4.06, 4.09, 4.40) HSQC(77.6) HMBC(87.5, 90.2)
98	α-Glucose	α-Glc	CH-1	5.26	*d*:3.79	COSY(3.56) TOCSY(3.44, 3.75, 3.86) HSQC(94.9) HMBC(74.2, 75.5)
99	Uracil		CH-6	5.8	*d*: 7.58	HSQC (103.8) HMDB (146.3)
100	Inosine	Ino	CH-1 (Rib)	6.09	*d*: 5.65	COSY(4.77) HSQC(91.2)
101	Inosine 5'phosphate	IMP	CH-1 (Rib)	6.13	*d*: 5.42	COSY(4.79) HSQC(90.3)
102	Fumarate		α,β CH=CH	6.54	*s*	HSQC (138.3)
103	Tyramine	Tyrm	CH-3,5	6.83	*d*:8.59	COSY(7.10) HSQC(118.3)
104	Tyrosine	Tyr	CH-3,5	6.86	*d*:8.47	COSY(7.16) HSQC(118.6) HMBC(129.4, 157.7)
105	Tyramine	Tyrm	CH-2,6	7.1	*m*	COSY(6.81) HSQC(133.42)

10<br>6	Tyrosine	Tyr	CH-2,6	7.14		COSY(6.86) HSQC(133.73) HMBC(157.7)
10<br>7	Anserine	Ans	CH-5 (His)	7.16	*s*	COSY(8.36) TOCSY(3.08, 3.25) HSQC(122.8)
10<br>8	Carnosine	Car	CH-5 (His)	7.17	*s*	COSY(8.30) TOCSY(3.08, 3.23) HSQC(119.8)
10<br>9	Phenylalanine	Phe	CH-2,6	7.32	*d*: 6.97	COSY(7.41) HSQC(132.3)
11<br>0	Phenylalanine	Phe	CH-4	7.35	*t*: 7.40	COSY(7.41) HSQC(130.5)
11<br>1	Phenylalanine	Phe	CH-3,5	7.41	*t*: 7.60	COSY(7.32, 7.35) HSQC(131.9)
11<br>2	uracil		CH-5	7.55	*d*: 7.73	HSQC (146.3) HMDB (103.8)
11<br>3	Nicotinic acid	NA	CH-5	7.59	*dd*: 8.00/5.02	COSY(8.23, 8.70) TOCSY(8.94) HSQC(127.1) HMBC(131.9, 154.6)
11<br>4	Inosine 5'phosphate	IMP	CH-8 (purin)	8.23	*s*	HSQC(149.2) HMBC(151.5, 161.3)
11<br>5	Nicotinic acid	NA	CH-4	8.23		COSY(7.59) TOCSY(8.86, 8.92) HSQC(139.4) HMBC(151.4, 161.4)
11<br>6	Carnosine	Car	CH-2 (His)	8.3	*s*	COSY(7.17) HSQC(137.0)
11<br>7	Anserine	Ans	CH-2 (His)	8.36	*s*	COSY(7.16) HSQC(139.0)
11<br>8	Inosine 5'phosphate	IMP	CH-2 (purin)	8.56	*s*	HSQC(142.7) HMBC(90.3, 126.2, 151.5, 161.3, 164.8)
11<br>9	Nicotinic acid	NA	CH-6	8.7	*dd*: 1.52/4.94	COSY(7.59) TOCSY(8.92) HSQC(154.8) HMBC(127.1, 132.0, 139.4, 150.4)
12<br>0	Nicotinic acid	NA	CH-2	8.92	*d*: 1.70	TOCSY(7.59, 8.23, 8.70) HSQC(150.4) HMBC(132.0, 139.4, 154.8)

[1] Represent peak splitting: *s*, singlet; *d*, doublet; *t*, triplet; *q*, quartet; *m*, multiplet; *dd*, double of doublets.
